# Elevated morbidity and mortality in patients with chronic idiopathic hypophosphatemia: a nationwide cohort study

**DOI:** 10.3389/fendo.2023.1229750

**Published:** 2023-08-10

**Authors:** Kyoung Jin Kim, Ji Eun Song, Ji Hyun Kim, Namki Hong, Sin Gon Kim, Juneyoung Lee, Yumie Rhee

**Affiliations:** ^1^ Division of Endocrinology and Metabolism, Department of Internal Medicine, Korea University College of Medicine, Seoul, Republic of Korea; ^2^ Department of Biostatistics, Korea University College of Medicine, Seoul, Republic of Korea; ^3^ Department of Internal Medicine, Endocrine Research Institute, Yonsei University College of Medicine, Seoul, Republic of Korea

**Keywords:** epidemiology, comorbidity, mortality, nationwide study, hypophosphatemia

## Abstract

**Background:**

Chronic idiopathic hypophosphatemia (CIH) induced by X-linked hypophosphatemic rickets or tumor-induced osteomalacia is a rare inherited or acquired disorder. However, due to its rarity, little is known about the epidemiology and natural course of CIH. Therefore, we aimed to identify the prevalence and long-term health outcomes of CIH patients.

**Methods:**

Using the Korean Health Insurance Review and Assessment claims database, we evaluated the incidence of hypophosphatemia initially diagnosed from 2003 to 2018. After excluding secondary conditions that could change serum phosphorus levels, we identified 154 patients (76 men and 78 women) with non-secondary and non-renal hypophosphatemia. These hypophosphatemic patients were compared at a ratio of 1:10 with age-, sex-, and index-year-matched controls (n = 1,540).

**Results:**

In the distribution of age at diagnosis, a large peak was observed in patients aged 1–4 years and small peaks were observed in ages from 40–70 years. The age-standardized incidence rate showed non-statistically significant trend from 0.24 per 1,000,000 persons in 2003 to 0.30 in 2018. Hypophosphatemic patients had a higher risk of any complication (adjusted hazard ratio [aHR], 2.17; 95% confidence interval [CI], 1.67–2.69) including cardiovascular outcomes, chronic kidney disease, hyperparathyroidism, osteoporotic fractures, periodontitis, and depression. Hypophosphatemic patients also had higher risks of mortality and hospitalization than the controls (aHR, 3.26; 95% CI, 1.83–5.81; and aHR, 2.49; 95% CI, 1.97–3.16, respectively).

**Conclusion:**

This first nationwide study of CIH in South Korea found a bimodal age distribution and no sex differences among patients. Hypophosphatemic patients had higher risks of complications, mortality, and hospitalization compared to age- and sex-matched controls.

## 1 Introduction

Phosphate is the most abundant anion, consisting of phospholipids and other intracellular compounds (1). Approximately 85% of total body phosphate is localized in the bones and teeth, 14% in the cells, and only 1% in the serum and extracellular fluids (2). It helps in energy storage and metabolism, regulates many coenzymes and oxygen transport, and performs other important biological functions for the immune and clotting systems (3). Therefore, its depletion can cause a broad spectrum of complications, including musculoskeletal, cardiovascular, neurologic, and hematologic disorders, through decreased intracellular adenosine triphosphate (ATP) and 2,3-diphosphoglycerate (2,3-DPG) (4). However, studies on the prognosis and long-term adverse events of chronic hypophosphatemia are limited.

Even though phosphate homeostasis is finely mediated by three main regulators such as vitamin D, parathyroid hormone (PTH), and fibroblast growth factor 23 (FGF-23), hypophosphatemia (<0.80 mmol/L or <2.48 mg/dL) can occur owing to decreased intestinal absorption, internal redistribution, and increased renal loss (5). Among them, FGF-23-related hypophosphatemic diseases, where elevated levels of FGF-23 inhibit proximal tubular phosphate reabsorption and intestinal phosphate absorption by decreasing serum 1,25-dihydroxyvitamin D (1,25D) are rare, including X-linked hypophosphatemic rickets (XLH) and tumor-induced osteomalacia (TIO) (6, 7). XLH is a form of heritable rickets caused by mutations in the *PHEX* gene (8, 9). TIO is an acquired paraneoplastic ricket induced by small, slowly growing tumors of mesenchymal origin or other rare tumors (10). Unfortunately, there have not been many studies or guidelines on either disease; thus, delayed diagnosis and treatment can contribute to increased risks of adverse outcomes and socioeconomic burdens (4).

Notably, epidemiological studies on chronic hypophosphatemia are still limited worldwide, with a focus primarily on the pediatric population and little emphasis on its long-term prognosis in adulthood (11). Furthermore, although burosumab, a recombinant human IgG1 monoclonal antibody against FGF-23, has been approved as a treatment for hypophosphatemic rickets in children and adults, patients in Korea are less likely to participate in randomized controlled trials than other ethnicities owing to a lack of epidemiologic research in the country (12).

Therefore, using the well-established Korean National Health Insurance Service (NHIS) database, this study aimed to explore the epidemiology of chronic idiopathic hypophosphatemia (CIH) in South Korea and investigated related complications and prognoses compared to the general population, regardless of exact etiologies.

## 2 Materials and methods

### 2.1 Data source

This study is based on the Korean NHIS database, which includes information on 51.5 million inhabitants in South Korea from 2002 and covers approximately 97% of the Korean population to facilitate reimbursements (13). This database contains information from 2002 to 2019, including demographics, hospitalization data, diagnostic codes (the International Classification of Disease, 10^th^ revision [ICD-10]), prescription claim records (such as medical procedures and drugs prescribed), and death records submitted to the NHIS by institutions. Additionally, we used specific diagnostic codes starting with the letter “V,” which are assigned to patients with rare, intractable diseases (RID). This code is quite reliable but limited because physicians are required to manually input diagnostic criteria when registering patients, with the primary objective of mitigating the burden of medical costs. We analyzed data from 2003 to 2018, with a 1-year washout period. The Institutional Review Board of Severance Hospital (IRB No. 2020-1106-002) approved the study protocol. The requirement for informed consent was waived because all data were anonymized.

### 2.2 Study population

Owing to the absence of specific ICD-10 codes for XLH and TIO, patients with CIH were defined as those who (1) had disorders of phosphorus metabolism and phosphatases (E83.3) as a main diagnosis or sub-diagnosis at least twice during the index year or had a health insurance benefit extension policy code (RID code) for disorders of phosphorus metabolism and phosphatases (V189) at least once between 2003 and 2018 and (2) were prescribed active vitamin D (calcitriol or alfacalcidol) within 6 months under the above-mentioned diagnostic codes. Patients who (1) had undergone tenofovir or adefovir treatment for >6 months during the study period; (2) had any diagnostic code for Fanconi syndrome or hypoparathyroidism during the study period; or (3) had any diagnostic code for hyperparathyroidism, disorders resulting from impaired renal tubular function, chronic kidney disease (CKD), or thyroidectomy due to thyroid cancer were excluded from the study to rule out secondary conditions that could have caused hypophosphatemia as a complication. This operational definition of CIH was validated using our institution’s database (sensitivity, 77.8%; positive predictive value, 100%).

We established a control group to represent the general population and analyze the risk of complications and mortality owing to CIH. Patients who did not meet any criteria for hypophosphatemia during the same period were regarded as the control group. Patients with hypophosphatemia were compared with their age-, sex-, and index year-matched controls in the ratio of 1:10. The index date was established as the time when the relevant diagnostic code (E83.3 or V189) was first assigned to CIH groups that met the inclusion and exclusion criteria.

### 2.3 Study outcomes and covariates

Any diagnosis of a complication 1-year after the index date was considered an incidental episode in both groups. Using ICD-10 codes, the following complications were chosen: cardiovascular events (non-fatal myocardial infarction, non-fatal stroke, heart failure, and arrhythmia), renal events (renal stones and CKD), hyperparathyroidism, fracture events (major osteoporotic fracture [MOF]; hip fracture; and vertebral, humeral, and wrist fractures), periodontitis, enthesopathy, and mental and behavior disorders (depression, bipolar disease, and anxiety) (**Supplementary Table S1**).

Comorbidities such as diabetes, hypertension, and dyslipidemia were defined by the presence of ICD-10 diagnostic codes with medical claims for at least two principal or secondary diagnoses and prescription medication use within 1 year before or after the index date (**Supplementary Table S1**). Based on the total amount of national health insurance premiums paid by insured individuals, we divided patients into three groups: the lowest 30%, the middle 40%, and the highest 30%.

### 2.4 Statistical analysis

Data are presented as the mean ± standard deviation for continuous variables or as numbers (%) for categorical variables. The age-standardized incidence rates of hypophosphatemia were calculated by dividing the number of cases in a specific age group by the corresponding age-specific national population. The Kaplan–Meier analysis and log-rank tests were performed to evaluate the relative hazard for mortality and hospitalization in patients with chronic hypophosphatemia compared to their controls; the relative hazards are presented as the hazard ratio (HR) and its 95% confidence interval (CI). A stratified Cox proportional hazard regression analysis was also used to evaluate the adjusted HRs of complications that developed 1 year after the index year. Confounders, including socioeconomic status and comorbidities such as diabetes, hypertension, and dyslipidemia were further adjusted. A P-value of <0.05 was considered statistically significant. SAS software version 9.4 (SAS Institute Inc., Cary, NC, USA) was used to conduct all statistical analyses.

## 3 Results

### 3.1 Epidemiology of chronic hypophosphatemia

**Figure 1:**
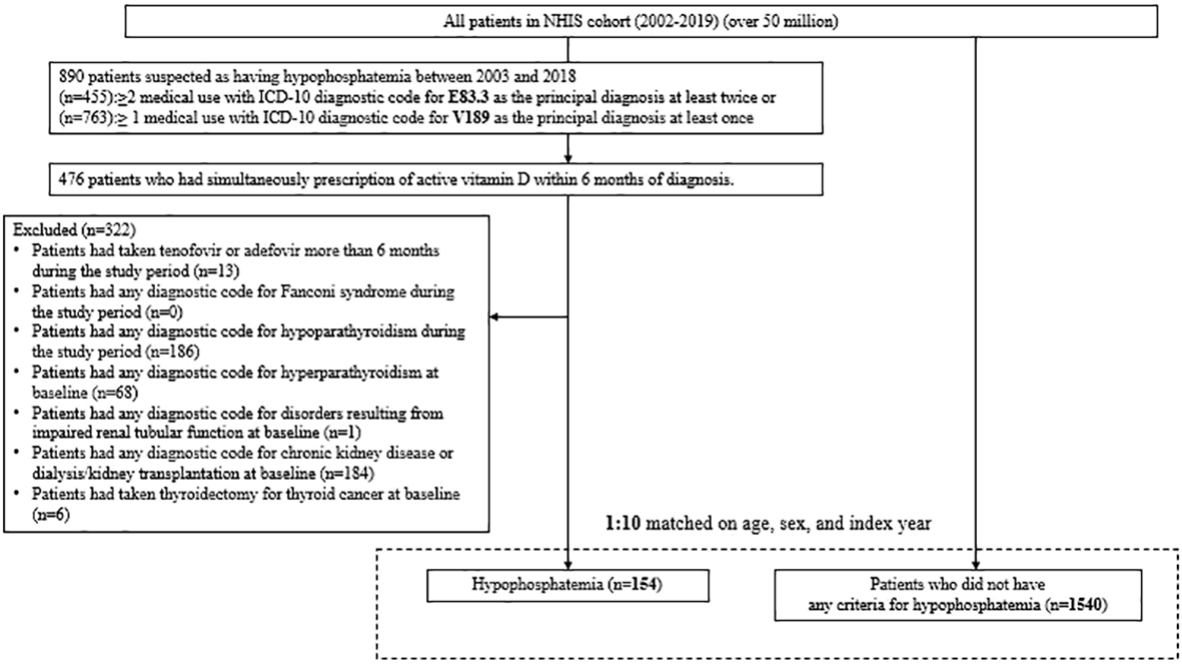
Flow diagram of participant selection.

**Figure 2:**
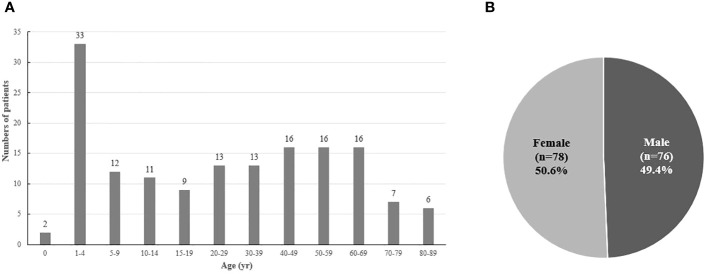
**(A)** Age at diagnosis and **(B)** sex distribution of patients with hypophosphatemia.

**Figure 3:**
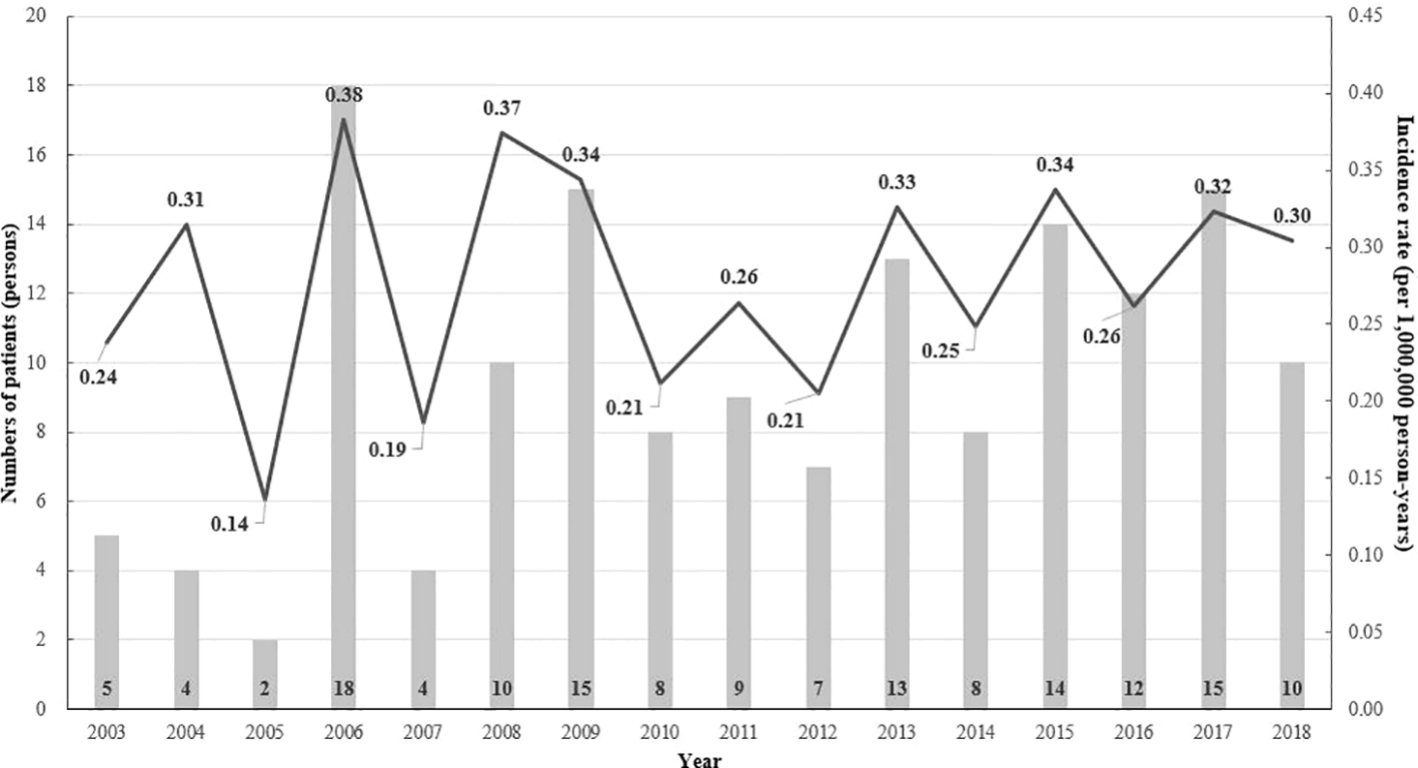
The annual age-standardized event rate (per 1 million) and the number of patients with hypophosphatemia by age and the index year (2003–2017).

From 2003 to 2018, there were 154 patients with CIH in Korea (**Figure 1**). After 1:10 age-, sex-, and index-year matching, the baseline characteristics of both the case and control groups are described in **Table 1**. The mean age was 31.5 ± 26.1 years, with double peaks in prevalence: the first peak between the 1–4 years of age group and the second peak between the 40–70 years of age group (**Table 1**, **Figure 2**). There was no difference with respect to sex in the prevalence, with a male: female ratio of 1:1.03 (men, n = 76 [49.4%]; women, n = 78 [50.6%]). Compared with controls, patients with CIH were more likely to have comorbidities, such as diabetes mellitus, hypertension, dyslipidemia, and vitamin D deficiency, and use more calcium and vitamin D supplements (**Table 1**). The overall age-standardized incidence rate of hypophosphatemia was 0.28 cases per 1,000,000 person-years, showing a stationary trend during the follow-up period, from 0.24 per 1,000,000 persons in 2003 to 0.30 per 1,000,000 persons in 2018 (**Figure 3**).

**Table 1:** Baseline characteristics of patients with chronic hypophosphatemia (n = 154).

Variables	Case			Control			P-value^*^
	Total (n = 154)	Men (n = 76)	Women (n = 78)	Total (n = 1540)	Men (n = 760)	Women (n = 780)	
Age at baseline, years, mean (SD)	31.5 (26.1)	32.6 (25.6)	30.4 (26.6)	31.5 (26.0)	32.6 (25.5)	30.4 (26.4)	1.00
Age group, n (%)							1.00
0	2 (1.3)	1 (1.3)	1 (1.3)	20 (1.3)	10 (1.3)	10 (1.3)	
1–4	33 (22.4)	17 (22.4)	16 (20.5)	330 (21.4)	170 (22.4)	160 (20.5)	
5–9	12 (7.8)	4 (5.3)	8 (10.3)	120 (7.8)	40 (5.3)	80 (10.3)	
10–14	11 (7.1)	3 (4.0)	8 (10.3)	110 (7.1)	30 (4.0)	80 (10.3)	
15–19	9 (5.8)	4 (5.3)	5 (6.4)	90 (5.8)	40 (5.3)	50 (6.4)	
20–29	13 (8.4)	8 (10.5)	5 (6.4)	130 (8.4)	80 (10.5)	50 (6.4)	
30–39	13 (8.4)	4 (5.3)	8 (10.3)	130 (8.4)	50 (6.6)	80 (10.3)	
40–49	16 (10.4)	12 (15.8)	4 (5.1)	160 (10.4)	120 (15.8)	40 (5.1)	
50–59	16 (10.4)	8 (10.5)	8 (10.3)	160 (10.4)	80 (10.5)	80 (10.3)	
60–69	16 (10.4)	8 (10.5)	8 (10.3)	160 (10.4)	80 (10.5)	80 (10.3)	
70–85	13 (8.4)	6 (7.9)	7 (9.0)	130 (8.5)	60 (7.9)	70 (9.0)	
Socioeconomic status							0.12
Lowest	47 (30.5)	23 (31.1)	24 (31.6)	377 (25.3)	196 (26.5)	181 (23.9)	
Middle	48 (32.0)	25 (33.8)	23 (30.3)	420 (28.1)	217 (29.4)	203 (26.8)	
Highest	55 (36.7)	26 (35.1)	29 (38.7)	700 (46.9)	326 (44.1)	374 (49.3)	
Missing	4 (2.6)	2 (2.6)	2 (2.6)	43 (2.8)	21 (2.8)	22 (2.8)	
Comorbidities							
Diabetes	16 (10.39)	10 (13.16)	6 (7.69)	39 (2.53)	28 (3.68)	11 (1.41)	<0.01
Hypertension	29 (18.83)	19 (25)	10 (12.82)	128 (8.31)	58 (7.63)	70 (8.97)	<0.01
Dyslipidemia	19 (12.34)	11 (14.47)	8 (10.26)	115 (7.47)	45 (5.92)	70 (8.97)	0.03
Concurrent medications							
Calcium supplementation	83 (53.90)	44 (57.89)	39 (50.00)	145 (9.42)	35 (4.61)	110 (14.10)	<0.01
Vitamin D (cholecalciferol, ergocalciferol)	55 (35.71)	23 (30.26)	32 (41.03)	127 (8.25)	27 (3.55)	100 (12.82)	<0.01
Active vitamin D (calcitriol, alfacalcidol)	151 (98.1)	74 (97.4)	77 (98.7)	13 (0.8)	4 (0.5)	9 (1.2)	<0.01

Data are shown as the mean ± SD or n (%).

SD, standard deviation.

^*^P-value for total case group vs. matched control group.

### 3.2 Risks of long-term adverse events in patients with hypophosphatemia

During a median follow-up period of 3.5 years (interquartile ranges, 1.9–7.1 years), the prevalence of any complication was higher in the CIH group than in the control group (72.5 vs. 60.7%; aHR 2.17; 95% CI, 1.67–2.69) (**Table 2**). Considering cause-specific complications, hypophosphatemic patients had higher risks of composite cardiovascular complications (aHR, 2.02; 95% CI, 1.16–3.51). Particularly, an elevated risk of heart failure was observed in the CIH group, independent of other confounding factors (aHR, 6.15; 95% CI, 2.15–17.58). Additionally, the risks of CKD and hyperparathyroidism were remarkably higher in the CIH group than in the control group (aHR, 16.87 vs. 121.19; 95% CI, 7.60–37.43 vs. 28.24–520.11). The risks of MOF and hip fracture were higher in the CIH group than in the control group (aHR, 2.27 vs. 3.83; 95% CI, 1.20–4.28 vs. 1.12–13.10). The risk of periodontitis was also higher in the CIH group than in the control group (aHR, 6.98; 95% CI, 1.66–29.35). Furthermore, hypophosphatemic patients had a higher risk of depression than their controls (aHR, 1.71; 95% CI, 1.04–2.83). However, there were no differences in the risks of renal stones, enthesopathy, and others between the two groups. The results of the subgroup analysis show that most complications develop in people over the age of 20, although the data are not shown.

**Table 2:** Complications of patients with chronic hypophosphatemia (n = 154).

	Case (n = 154)	Controls (n = 1540)	Unadjusted HR (95% CI)	P-value	Adjusted HR* (95% CI)	P-value
Any complications	127 (82.5)	935 (60.7)	2.74 (2.22–3.39)	<0.01	2.17 (1.67–2.69)	<0.01
Cardiovascular complications						
Composite CVD	20 (13.0)	98 (6.4)	2.80 (1.69–4.63)	<0.01	2.02 (1.16–3.51)	0.01
Non-fatal MI	3 (1.3)	13 (0.8)	3.92 (1.01–15.19)	0.048	1.61 (0.29–8.83)	0.59
Non-fatal stroke	6 (3.9)	25 (1.6)	3.06 (1.22–7.72)	0.02	2.65 (0.95–7.39)	0.06
Heart failure	9 (5.8)	26 (1.7)	8.41 (3.41–20.70)	<0.01	6.15 (2.15–17.58)	<0.01
Arrhythmia	6 (3.9)	58 (3.8)	1.11 (0.48–2.60)	0.80	0.98 (0.40–2.39)	0.96
Renal complications						
Renal stones	4 (2.6)	27 (1.8)	1.93 (0.66–5.64)	0.23	2.18 (0.70–6.80)	0.18
Chronic kidney disease	25 (16.2)	19 (1.2)	20.07 (10.08–39.96)	<0.01	16.87 (7.60–37.43)	<0.01
Hyperparathyroidism	24 (15.6)	2 (0.1)	114.40 (27.03–484.21)	<0.01	121.19 (28.24–520.11)	<0.01
Fracture complication						
Major osteoporotic fracture	13 (2.6)	71 (4.6)	2.41 (1.31–4.44)	<0.01	2.27 (1.20–4.28)	0.01
Hip fracture	4 (2.6)	9 (0.6)	8.93 (2.23–35.80)	<0.01	3.83 (1.12–13.10)	0.03
Vertebral fracture	5 (3.3)	33 (2.1)	1.97 (0.75–5.19)	0.17	1.75 (0.64–4.83)	0.28
Humeral or wrist fractures	4 (2.6)	34 (2.2)	1.24 (0.44–3.53)	0.68	1.20 (0.40–3.59)	0.75
Periodontitis	6 (3.9)	13 (0.8)	9.66 (3.11–29.95)	<0.01	6.98 (1.66–29.35)	0.01
Enthesopathy	16 (10.4)	129 (8.4)	1.46 (0.86–2.47)	0.16	1.60 (0.93–2.77)	0.09
Mental and behavior disorders						
Depression	20 (13.0)	154 (10.0)	1.72 (1.06–2.77)	0.03	1.71 (1.04–2.83)	0.04
Bipolar	2 (1.3)	31 (2.0)	0.76 (0.18–3.21)	0.71	0.79 (0.18–3.42)	0.74
Anxiety	27 (17.5)	210 (13.6)	1.61 (1.07–2.44)	0.02	1.46 (0.95–2.24)	0.08

CI, confidence interval; CVD, cardiovascular disease; HR, hazard ratio; MI, myocardial infarction. *Adjusted for socioeconomic status and comorbidities (diabetes, hypertension, and dyslipidemia). The adjusted HR (95% CI) and P-values were obtained using stratified Cox proportional hazards regression analysis.

### 3.3 Mortality and hospitalization outcomes

**Figure 4:**
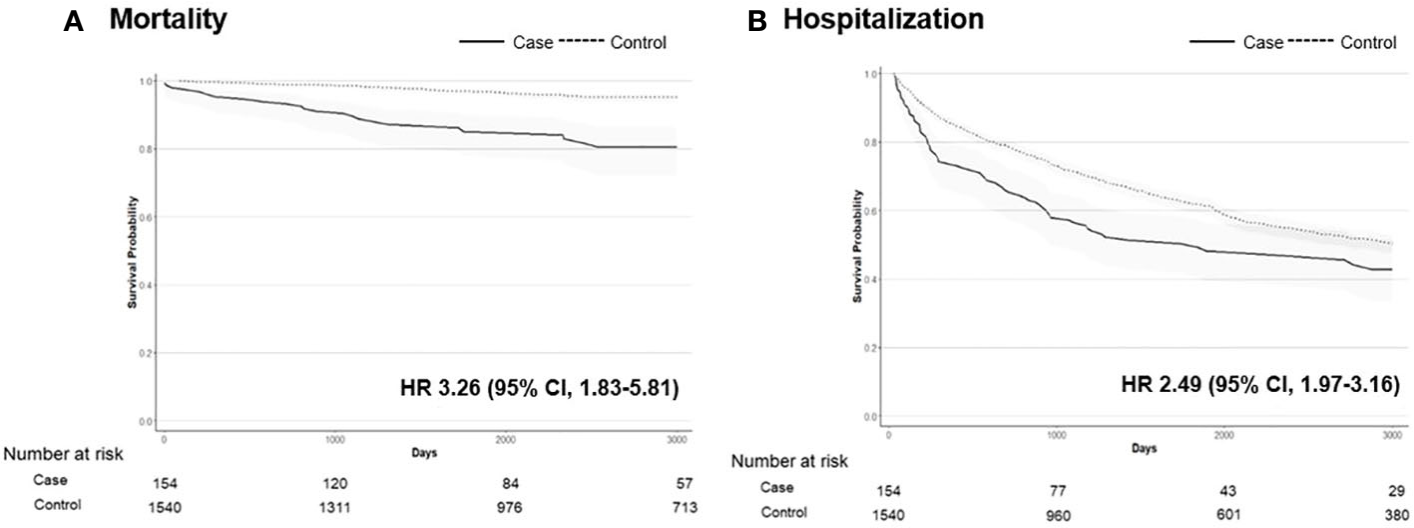
The Kaplan–Meier survival curve of mortality **(A)** and hospitalization **(B)** in patients with hypophosphatemia (n = 154) vs. controls (n = 1540). Hazard ratios (HRs) with 95% confidence intervals (CIs) are shown.

In the hypophosphatemic group, there were 17 (11.0%) deaths at a median age of 59.5 years, which equates to an incidence rate of 1.23 per 1,000 person-years. In the control group, there were 80 (5.2%) deaths at a median age of 73.6 years, with an incidence rate of 0.55 per 1,000 person-years. The risk of all-cause mortality in the CIH group was significantly higher than that in the control group (HR, 3.26; 95% CI, 1.83–5.81) (**Figure 4A**). The risk of hospitalization owing to any cause was also higher in the CIH group than in the control group (HR, 2.49; 95% CI, 1.97–3.16) (**Figure 4B**).

## 4 Discussion

This was the first nationwide population-based epidemiological study of CIH in South Korea, providing an overall age-standardized incidence rate of 0.28 cases per million. We observed a bimodal age distribution and no sex difference in the hypophosphatemic group. The complication risks for composite cardiovascular outcomes, heart failure, CKD, hyperparathyroidism, MOF, hip fracture, periodontitis, and depression were higher in hypophosphatemic patients than in age- and sex-matched controls. We also observed that hypophosphatemic patients had higher risks of mortality and hospitalization than the controls.

Phosphate homeostasis is maintained by a harmonized hormonal triad consisting of FGF23, PTH, and 1,25D (14). FGF23, primarily secreted by osteocytes, plays a critical role in this regulatory system (15). Prolonged increases in extracellular fluid phosphate levels stimulate FGF23 production, which subsequently reduces renal phosphate reabsorption, effectively counteracting hyperphosphatemia (16). Concurrently, a decrease in renal 1,25D production reduces phosphate absorption in the gastrointestinal tract (17). Overall, this endocrine regulation is to prevent hyperphosphatemia and maintain normophosphatemia. In contrast, lower extracellular phosphate concentrations lead to FGF23 downregulation, resulting in increased renal phosphate reabsorption and intestinal absorption, counteracting hypophosphatemia (18). FGF23 primarily modulates phosphate metabolism, while both PTH and 1,25D regulate phosphate and calcium homeostasis. These hormones exhibit reciprocal modulation of each other’s secretion. Hypophosphatemia arising from an intrinsic increase in FGF23 secretion is characterized by reduced phosphate reabsorption, low 1,25D, and high-normal PTH (19). Within the chronic idiopathic hypophosphatemia spectrum, two conditions, X-linked hypophosphatemia (XLH) and tumor-induced osteomalacia (TIO), are notably distinct (20).

Although a few epidemiologic studies have been conducted in other countries, their results should be interpreted with caution because of differences in their cohort populations. In southern Denmark, 112 patients with nutritional rickets were identified, indicating that the prevalence of hypophosphatemic rickets was 4.3 per million (21). A recent population-based study conducted in Norway identified only 28 patients with hereditary hypophosphatemia, among whom 21 children were confirmed to have XLH with a prevalence of 16.6 per million (22). Furthermore, an analysis of real-world data from the United Kingdom showed an increasing trend in the prevalence of XLH, from 3.0 per million in 1995–1999 to 8.1 per million in 2012–2016 by using the conservative definition (23). In the case of TIO, the incidence had been reported to be much lower than that of XLH, which estimates that the incidence of TIO in Denmark was below 0.13 per 100,000 person-years (24). In Japan, the first epidemiologic study regarding FGF23-related hypophosphatemic diseases, including both XLH and TIO, revealed that the estimated annual incidence rate was 117 cases (11). In this study, we could not distinguish each cause of CIH because specific ICD-10 codes for either XLH or TIO do not exist as diagnostic gold standards and orphanet codes are also not available in this cohort. However, we reported a total of 154 patients with CIH, providing an overall incidence rate of 0.28 per million. The etiology behind the lower incidence rate of CIH in Korea in comparison to other countries remains unclear. Setting operational definitions for CIH that are too conservative to accurately detect cases might contribute to missing more cases. Nevertheless, this was the first epidemiological study in South Korea on hypophosphatemia, possibly including XLH and TIO. Further nationwide cohort studies are needed to accurately identify the epidemiology of both diseases.

Few studies have reported the risks of long-term complications in patients with CIH. A Norwegian study reported that patients with hereditary hypophosphatemia, especially those who initiated early treatment and took higher daily doses of phosphate, were at high risk of developing nephrocalcinosis (22). However, our findings showed that the risk of renal stones was not increased in patients with CIH. Although higher risks of CKD and hyperparathyroidism, both of which are associated with renal stones, were observed in patients with CIH compared to their controls, monitoring of renal stones might not be performed owing to a lack of awareness of these diseases (4). Disruption of the physiological regulation of PTH secretion in CIH patients has been described in previous studies, although the interactions between phosphate, FGF23, and PTH remain unclear (1). Additionally, oral phosphate supplementation as a treatment for CIH can cause secondary hyperparathyroidism (25), which was also observed in our study. Interestingly, our study showed an increased risk of heart failure (approximately 6.1-fold) in patients with CIH compared with controls. The decline in 2,3-DPG in erythrocytes and the depletion of ATP in myocardial cells could both be contributing factors to hypophosphatemia-induced cardiomyopathy (26). Musculoskeletal complications related to hypophosphatemia included osteomalacia (MOF and hip fracture) and dental anomalies (periodontitis), which were also observed in our study. Regarding the higher risks of hip fractures than other sites, lifelong hypophosphatemia induces deterioration in the microarchitecture of the cortical bone, which causes osteomalacia in combination with skeletal deformities from childhood (27–29). Herrou et al. also found that adult XLH patients have a high prevalence of dental diseases because of the severity of osteomalacia (30). The association between hypophosphatemia and mood disorders has not been presented in previous studies. Suffering from chronic pain and taking multiple drugs lifelong can cause depression in CIH patients. Further studies are needed to clarify the direct association between CIH and psychiatric disorders. This higher risk of complications might contribute to an increased risk of hospitalization.

Additionally, we identified a higher risk of mortality in CIH patients. The association between hypophosphatemia and mortality among hospitalized patients has been consistently shown; however, the possible cause has never been clarified (31). According to the results of the study conducted in the United Kingdom, an unexpected increase in mortality in later life was observed in patients with XLH (23). The mechanism underlying the association between CIH and mortality is unknown; nonetheless, a higher risk of complications indirectly induces higher mortality. Furthermore, elevated levels of FGF23 are associated with increased vascular calcification and higher mortality, although future studies are needed to confirm these associations (32).

To our knowledge, this is the first Korean study to investigate the epidemiology and long-term events in CIH patients of all ages. Furthermore, our analysis of the risks of different complications in CIH patients can enrich the scope of research in this field. However, our study had several limitations. First, we used an operational definition for CIH based on diagnostic and prescription codes rather than the results of genetic testing for XLH and pathological findings for TIO. Additionally, the diagnosis of CIH may not be accurate since this cohort did not have data on phosphate and FGF23 for evaluating the state of hypophosphatemia. However, we have attempted to exclude patient groups, such as those with CKD or hyperparathyroidism that could develop the condition secondarily as possible to focus on the specific patient group with CIH. Second, the number of outcomes was small, which might limit the statistical analysis. Lastly, detection bias might contribute to more complications in patients compared to their controls.

In conclusion, our nationwide population-based study showed a small number of CIH patients in South Korea, which may be an underestimation. However, the risks of long-term adverse events and mortality were higher in these patients than in the control group. Therefore, early detection and an ideal treatment approach are needed to improve long-term adverse events of patients. Moreover, considering the limited information owing to its rarity, further studies with constant attention are required.

## Data availability statement

Publicly available datasets were analyzed in this study. This data can be found here: https://nhiss.nhis.or.kr/bd/ab/bdaba021eng.do. (The Korean National Health Insurance Service).

## Ethics statement

The studies involving humans were approved by Institutional Review Board of Severance Hospital. The studies were conducted in accordance with the local legislation and institutional requirements. The ethics committee/institutional review board waived the requirement of written informed consent for participation from the participants or the participants’ legal guardians/next of kin because The requirement of informed consent was waived because All data were anonymized.

## Author contributions

KK, NH and YR conceived and designed the study. KK, JS, JK, JL, and YR analyzed and verified the data. KK and YR wrote the manuscript. NH, SK, and JLperformed the critical reviews. All the authors contributed to the review and revision of the manuscript. The corresponding author attests that all listed authors meet the authorship criteria and that no others meeting the criteria have been omitted. All authors contributed to the article and approved the submitted version.

## References

[1] Manghat P Sodi R Swaminathan R. Phosphate homeostasis and disorders. Ann Clin Biochem (2014) 51:631–56. doi: 10.1177/0004563214521399 24585932

[2] Huang X Jiang Y Xia W. FGF23 and phosphate wasting disorders. Bone Res (2013) 1:120–32. doi: 10.4248/BR201302002 PMC4472102 26273497

[3] Weisinger JR Bellorín-Font E. Magnesium and phosphorus. Lancet (1998) 352:391–6. doi: 10.1016/S0140-6736(97)10535-9 9717944

[4] Gaasbeek A Meinders AE. Hypophosphatemia: an update on its etiology and treatment. Am J Med (2005) 118:1094–101. doi: 10.1016/j.amjmed.2005.02.014 16194637

[5] Fukumoto S. Phosphate metabolism and vitamin D. Bonekey Rep (2014) 3:497. doi: 10.1038/bonekey.2013.231 24605214 PMC3944128

[6] Marcucci G Masi L Ferrarì S Haffner D Javaid MK Kamenický P. Phosphate wasting disorders in adults. Osteoporos Int (2018) 29:2369–87. doi: 10.1007/s00198-018-4618-2 30014155

[7] Fukumoto S. FGF23-related hypophosphatemic rickets/osteomalacia: diagnosis and new treatment. J Mol Endocrinol (2021) 66:R57–65. doi: 10.1530/JME-20-0089 33295878

[8] Trombetti A Al-Daghri N Brandi ML Cannata-Andía JB Cavalier E Chandran M. Interdisciplinary management of FGF23-related phosphate wasting syndromes: a Consensus Statement on the evaluation, diagnosis and care of patients with X-linked hypophosphataemia. Nat Rev Endocrinol (2022) 18:366–84. doi: 10.1038/s41574-022-00662-x 35484227

[9] Haffner D Emma F Eastwood DM Duplan MB Bacchetta J Schnabel D. Clinical practice recommendations for the diagnosis and management of X-linked hypophosphataemia. Nat Rev Nephrol (2019) 15:435–55. doi: 10.1038/s41581-019-0152-5 PMC7136170 31068690

[10] Minisola S Peacock M Fukumoto S Cipriani C Pepe J Tella SH. Tumour-induced osteomalacia. Nat Rev Dis Primers (2017) 3:17044. doi: 10.1038/nrdp.2017.44 28703220

[11] Endo I Fukumoto S Ozono K Namba N Inoue D Okazaki R. Nationwide survey of fibroblast growth factor 23 (FGF23)-related hypophosphatemic diseases in Japan: prevalence, biochemical data and treatment. Endocr J (2015) 62:811–6. doi: 10.1507/endocrj.EJ15-0275 26135520

[12] Schindeler A Biggin A Munns CF. Clinical evidence for the benefits of burosumab therapy for X-linked hypophosphatemia (XLH) and other conditions in adults and children. Front Endocrinol (Lausanne) (2020) 11:338. doi: 10.3389/fendo.2020.00338 32547492 PMC7271822

[13] Cheol Seong S Kim YY Khang YH Heon Park J Kang HJ Lee H. Data resource profile: the national health information database of the national health insurance service in South Korea. Int J Epidemiol (2017) 46:799–800. doi: 10.1093/ije/dyw253 27794523 PMC5837262

[14] Blau JE Collins MT. The PTH-Vitamin D-FGF23 axis. Rev Endocr Metab Disord (2015) 16:165–74. doi: 10.1007/s11154-015-9318-z 26296372

[15] Shimada T Hasegawa H Yamazaki Y Muto T Hino R Takeuchi Y. FGF-23 is a potent regulator of vitamin D metabolism and phosphate homeostasis. J Bone Miner Res (2004) 19:429–35. doi: 10.1359/JBMR.0301264 15040831

[16] Balani S Perwad F. Fibroblast growth factor 23 and phosphate homeostasis. Curr Opin Nephrol Hypertens (2019) 28:465–73. doi: 10.1097/MNH.0000000000000526 31335449

[17] Shimada T Kakitani M Yamazaki Y Hasegawa H Takeuchi Y Fujita T. Targeted ablation of Fgf23 demonstrates an essential physiological role of FGF23 in phosphate and vitamin D metabolism. J Clin Invest (2004) 113:561–8. doi: 10.1172/JCI19081 PMC338262 14966565

[18] Peacock M. Phosphate metabolism in health and disease. Calcif Tissue Int (2021) 108:3–15. doi: 10.1007/s00223-020-00686-3 32266417

[19] Zhang X Imel EA Ruppe MD Weber TJ Klausner MA Ito T. Pharmacokinetics and pharmacodynamics of a human monoclonal anti-FGF23 antibody (KRN23) in the first multiple ascending-dose trial treating adults with X-linked hypophosphatemia. J Clin Pharmacol (2016) 56:176–85. doi: 10.1002/jcph.570 PMC5042055 26073451

[20] Tebben PJ. Hypophosphatemia: A practical guide to evaluation and management. Endocr Pract (2022) 28:1091–9. doi: 10.1016/j.eprac.2022.07.005 35940468

[21] Beck-Nielsen SS Brock-Jacobsen B Gram J Brixen K Jensen TK. Incidence and prevalence of nutritional and hereditary rickets in southern Denmark. Eur J Endocrinol (2009) 160:491–7. doi: 10.1530/EJE-08-0818 19095780

[22] Rafaelsen S Johansson S Ræder H Bjerknes R. Hereditary hypophosphatemia in Norway: a retrospective population-based study of genotypes, phenotypes, and treatment complications. Eur J Endocrinol (2016) 174:125–36. doi: 10.1530/EJE-15-0515 PMC4674593 26543054

[23] Hawley S Shaw NJ Delmestri A Prieto-Alhambra D Cooper C Pinedo-Villanueva R. Prevalence and mortality of individuals with X-linked hypophosphatemia: A United Kingdom real-world data analysis. J Clin Endocrinol Metab (2020) 105:e871–8. doi: 10.1210/clinem/dgz203 PMC7025948 31730177

[24] Abrahamsen B Smith CD Minisola S. Epidemiology of tumor-induced osteomalacia in Denmark. Calcif Tissue Int (2021) 109:147–56. doi: 10.1007/s00223-021-00843-2 PMC8273058 33818653

[25] Takasugi S Akutsu M Nagata M. Oral phosphorus supplementation secondarily increases circulating fibroblast growth factor 23 levels at least partially via stimulation of parathyroid hormone secretion. J Nutr Sci Vitaminol (Tokyo) (2014) 60:140–4. doi: 10.3177/jnsv.60.140 24975224

[26] Ariyoshi N Nogi M Ando A Watanabe H Umekawa S. Hypophosphatemia-induced cardiomyopathy. Am J Med Sci (2016) 352:317–23. doi: 10.1016/j.amjms.2016.04.013 27650239

[27] Javaid MK Ward L Pinedo-Villanueva R Rylands AJ Williams A Insogna K. Musculoskeletal features in adults with X-linked hypophosphatemia: an analysis of clinical trial and survey data. J Clin Endocrinol Metab (2022) 107:e1249–e62. doi: 10.1210/clinem/dgab739 PMC8852215 34636401

[28] Lecoq AL Brandi ML Linglart A Kamenický P. Management of X-linked hypophosphatemia in adults. Metabolism (2020) 103s:154049. doi: 10.1016/j.metabol.2019.154049 31863781

[29] Tiefenbach M Scheel M Maier A Gehlen M Schwarz-Eywill M Werner M. Osteomalacia-Clinical aspects, diagnostics and treatment. Z Rheumatol (2018) 77:703–18. doi: 10.1007/s00393-018-0510-x 30097703

[30] Herrou J Picaud AS Lassalle L Pacot L Chaussain C Merzoug V. Prevalence of enthesopathies in adults with X-linked hypophosphatemia: analysis of risk factors. J Clin Endocrinol Metab (2022) 107:e224–35. doi: 10.1210/clinem/dgab580 34406383

[31] Brunelli SM Goldfarb S. Hypophosphatemia: clinical consequences and management. J Am Soc Nephrol (2007) 18:1999–2003. doi: 10.1681/ASN.2007020143 17568018

[32] Souma N Isakova T Lipiszko D Sacco RL Elkind MS DeRosa JT. Fibroblast growth factor 23 and cause-specific mortality in the general population: the Northern Manhattan study. J Clin Endocrinol Metab (2016) 101:3779–86. doi: 10.1210/jc.2016-2215 PMC5052338 27501282

