# Supplementary material for: Elevated morbidity and mortality in patients with chronic idiopathic hypophosphatemia: a nationwide cohort study
Source: Front Endocrinol (Lausanne). 2023 Aug 10;14:1229750. doi: 10.3389/fendo.2023.1229750 (PMC10448510; doi:10.3389/fendo.2023.1229750)
Supplement: Supplementary file 1 [file DataSheet_1.pdf]

Elevated Morbidity and Mortality in Patients with Chronic Idiopathic Hypophosphatemia: A Nationwide Cohort Study

Running title: Korean nationwide study of hypophosphatemia

Authors: Kyoung Jin Kim, Ji Eun Song, Ji Hyun Kim, Namki Hong, Sin Gon Kim,

Juneyoung Lee, Yumie Rhee

Supporting Information

Contents	Page
**Table S1.** Definitions and codes used for defining key conditions, comorbidities, drug treatments in this study	2-3

Supplemental Table S1. Definitions and codes used for defining key conditions, comorbidities, drug treatments in this study

	Definitions	ICD-10 Codes or conditions
**Comorbidities**		
Diabetes mellitus	Defined from diagnosis plus treatment (≥ 30 days) at least once	ICD-10 E10-E14<br>Treatment: various oral antidiabetics (alpha-glucosidase inhibitor, DPP-4 inhibitor, GLP1 agonist, meglitinide, metformin, SGLT2 inhibitor, sulfonylurea, thiazolidinedione) and insulin.
Hypertension	Defined from diagnosis plus treatment (≥ 30 days) at least once	ICD-10 I10<br>Treatment: various antihypertensive agents (ARB, ACE inhibitors, beta blockers, calcium channel blockers, alpha blockers, diuretics, and others)
Dyslipidemia	Defined from diagnosis plus treatment (≥ 30 days) at least once	ICD-10 78<br>Treatment: various lipid lowering agents (statins, fibrates)
Osteoporosis	Defined from prescription codes for any treatment received at least once	ICD-10 M80-M82<br>Treatment: bisphosphonate (alendronate, etidronate, ibandronate, risedronate, pamidronate, zoledronate), selective estrogen receptor modulator (bazedoxifene, raloxifene), or hormone replacement therapy (allylestrenol, chlormadinone, dienogest, diethylstilbestrol, dydrogesterone, estradiol, estrogen, ethynylestradiol, levonorgestrel, medroxyprogesterone)
**Complications**		
Cardiovascular complications		
Composite cardiovascular disease	Any event of non-fatal myocardial infarction, non-fatal stroke, hospitalization for heart failure or arrhythmia	
Non-fatal myocardial infarction	Defined from a diagnostic code of MI plus a procedure code of coronary artery angiography	ICD-10: I20–I25; claim for coronary revascularization (HA607, E0721, E0723, O1640, O1641, O1642, O1647, O1648, O1649, OA640, OA641, OA642, OA647, OA648, OA649, M6551, M6552, M6553, M6554, M6561, M6562, M6563, M6564, M6565, M6566, M6567, M6571, M6572)
Non-fatal stroke	Defined from a diagnostic code of stroke plus a procedure code of brain image	ICD-10: I63–I66; claims for brain image (HE101, HE102, HE135, HE136, HE201, HE202, HE235, HE236, HE301, HE302, HE501, HE502, HE535, HE536)
Heart failure	Defined from principal or first secondary admission diagnosis of heart failure	ICD-10: I50, I42, I43
Arrhythmia	Defined from diagnosis with one inpatient or two outpatient records	ICD-10: I44-I45, I47-I49
Renal complications		

Renal stones	Defined from diagnostic codes for at least 2 principal or secondary diagnosis	ICD-10: N20-N23
Chronic kidney disease	Defined from diagnosis or any procedure code for kidney replacement treatment	ICD-10: N18-N19, Z94.0<br>Claims for kidney replacement treatment (Z49.1-49.2, O7072, O7073-7034, O7071, O7072, R3280)
Hyperparathyroidism	Defined from diagnostic codes for at least 2 principal or secondary diagnosis	ICD-10: E21
Fracture complications		
Hip fracture	Defined from a diagnostic code of hip fracture	ICD-10: S72.0, S72.1
Vertebral fracture	Defined from a diagnostic code of vertebral fracture	ICD-10: S22.0, S22.1, S32.0, S32.7, T080, M48.4, M48.5, M49.5, M80.8
Humerus or wrist fracture	Defined from a diagnostic code of humerus or distal radius fracture	ICD-10: S42.2, S42.3, S52.5, S52.6
Chronic kidney disease	Defined from diagnosis or any procedure code for kidney replacement treatment	ICD-10: N18, N18.5, Z94.0<br>Claims for kidney replacement treatment (Z49.1-49.2, O7072, O7073-7034, O7071, O7072, R3280)
Periodontitis	Defined from diagnostic codes for at least 2 principal or secondary diagnosis	ICD-10: K05.2-K05.6
Enthesopathy	Defined from diagnostic codes for at least 2 principal or secondary diagnosis	ICD-10: M46, M76, M77
Cancer	Defined from diagnostic codes for at least 2 principal or secondary diagnosis	ICD-10: C00-C97
Mental and behavior disorders		
Depression	Defined from diagnostic codes for at least 2 principal or secondary diagnosis	ICD-10: F32-F33
Bipolar disease	Defined from diagnostic codes for at least 2 principal or secondary diagnosis	ICD-10: F30, F31, F34
Anxiety	Defined from diagnostic codes for at least 2 principal or secondary diagnosis	ICD-10: F40-F42

ICD-10, International Classification of Diseases, 10th revision.
